# A Narrative Review of Nutritional Malpractices, Motivational Drivers, and Consequences in Pregnant Women: Evidence from Recent Literature and Program Implications in Ethiopia

**DOI:** 10.1155/2021/5580039

**Published:** 2021-06-19

**Authors:** Gesessew Kibr

**Affiliations:** Department of Food and Nutritional Sciences, Faculty of Agriculture, Shambu Campus, Wollega University, P. O. Box: 38, Shambu, Ethiopia

## Abstract

Maternal nutrition is very important for the wellbeing of pregnant women, childbirth, and lactating women, which are crucial and meant for the wellbeing of a mother and newborn baby. This narrative review discusses nutritional malpractices, motivational drivers, and their consequences typically from Ethiopian pregnant women's context. Different studies (regarding less of study design and type) done among pregnant women (aged 15–49 years) by considering pregnancy-related outcomes and timing of nutritional malpractices were included mostly. Accordingly, taboos of healthy diets, craving for unhealthy foods (sweet, fat, raw, and salty/spicy foods), and nonfood items (soil, coffee residue, stone, and ash) were practiced majorly by the women. The birth difficulty, fetal head plastering, fetus discoloration, fetus burns, abortion, and abdominal cramp are the primary drivers of taboos of healthy diets. Hormonal change and social and nutrient-seeking behavior are the most prevalent drivers to the consumption of unhealthy foods. Additionally, personal interest, flavor, and color of items are important motivators to practice pica. Such pica practice hurts nutrient intake, absorption of iron/zinc, abdominal health, and diarrhea occurrence. Food taboos are high predictors of health disorders, such as intrauterine growth restriction, infection, bleeding, preeclampsia, stillbirth, early birth, low birth weight, retarded development of cognitive, and anemia. Craving and eating unhealthy foods were interconnected with chronic disease development (hypertension, diabetes, heart disease, and cancer), discomforts, preterm labor, preeclampsia, and intrauterine growth restriction in women. Additionally, it is also associated with stillbirth, low birth weight, obesity, birth defect/deficit, hypertension, cancer, diabetes, metabolic syndrome, renal disease, decreased fetal growth, behavioral change, heart failure, and poor cognitive development in the infant. Overall, these nutritional malpractices are significantly associated with many argumentative pregnancies as well as developmental consequences leading to the direction of infant and maternal mortality and morbidity. Therefore, urgent implementation of health and nutrition education programs considering food misconceptions and beliefs regarding pregnancy and use of ground-breaking ways to play down the negative and maximize potential positive dietary effects designed by the government of Ethiopia could also serve as a long-term solution to the problem.

## 1. Introduction

Malnutrition is a provision that results from inadequate intake of nutrients or inability to fully utilize the food eaten due to illness and causes further health problems [1]. Maternal health care and tolerable nutritional status are excellent for physical condition, improving women's work capacity, and maintaining the health of the child [2–5]. In developing countries, the underlying cause of malnutrition is highly prevalent and causes the death of children aged below five and women each year [2]. Balanced nutrition during pregnancy helps to improve birth outcomes and prevent the child from developing diseases such as heart disease and obesity later in life. Proper food and good nutrition are essential for survival, physical growth, mental development, performance and productivity, and health and wellbeing [6]. Unbalanced intrahousehold diet allocation, little nutritional intakes, frequent occurrence of infections, and pitiable care are among the most important causes of undernutrition, but food misconceptions and taboos can also supply drastically to the high levels of maternal malnutrition [7, 8]. Accordingly, nutritional malpractices in women have been acknowledged as solitary factors contributory to the malnutrition of women [9]. Physiological and psychological changes that occur during pregnancy also impact the possibility of exhibiting food cravings, aversions, nausea, and vomiting [10, 11]. These complications may not only cause discomfort during pregnancy but also interfere with the dietary intakes of the pregnant woman as well as causing serious problems [10].

The availability and preferences of food bind with intellectual traditions which are influential to affect the daily life at the time of pregnancy and the possibility of developing craving behaviors to different food items [11]. The relative contribution of these nutritional malpractices shows a discrepancy significantly by dietary practices, time of year, and geographical setting [12] and time, culture, norm, and religion [13]. With regard to food accessibility, the importance of the food environment is underscored [14–16]. The food environment that encompasses domains such as accessibility, availability, and affordability is an interface whereby individuals interact with the wider food system for food purchase and consumption [17]. Logically, individuals can only eat from the range of things that are available to them, and the availability of an adequate supply of healthy food has been consistently associated with a healthy diet [18]. The availability of fresh healthy foods has also been improved by the development of advanced agricultural technologies and transport systems that can provide cold chain equipment to preserve perishables. However, as these technologies are undeveloped in low-income settings, 40% of fresh food does not reach consumers due to postharvest loss [19, 20]. Another important dimension related to diet is affordability [18]. In low-income urban areas where availability is limited, only the economically better-off families manage to access a wide range of the recommended food groups [20, 21].

Despite sufficient access to markets, dietary choices are influenced by price [22]. The results from a multicountry study showed that individuals from low-income countries spend more than half of their income to meet dietary recommendations [23]. The high cost prevents poorer households from affording a nutritionally adequate diet [22]. This is more common in urban areas, where the only source of food for most of households is that which is purchased. Furthermore, less healthy alternatives are often inexpensive, have a longer shelf-life, require little preparation, and have an alluring taste, making them both convenient and desirable for the women who are responsible for purchasing and preparing food for the family [15, 24, 25].

However, accessibility and production of healthy food items are not fully guaranteed to improve women's nutritional status, and there is a need for particular consideration for food misconceptions and taboos that are identified with specific concern in the community [26]. Different recent studies from different parts of Ethiopia have largely reported cultural taboo: fruits and vegetables and cereals and salty food [27–31]; dairy foods such as yogurt, milk, and cheese [26, 30, 32]; and egg, fatty meat, and honey [29, 30, 32].

Food cravings and aversions are extremely familiar during the pregnancy period; however, the causes underlying such nutritional malpractices are not well stated [33]. Distinctively, craving is a desire to eat food items [34] and nonfood items extremely [35–38]. The phenomenon of food craving during pregnancy was investigated all over the world in the past few decades and about 60–85% of women were reported [39]. From oily fried and chats to ice cream at midnight, pregnant women have some pretty crazy food cravings during pregnancy and it is a common experience every pregnant woman has especially for unhealthy foods. Additionally, eating of the craved food items, which generally include junk foods such as sweet, fat, salty, and spicy food items, may be a response to deficiencies in important nutrients [33], which hurts pregnancy outcomes [40, 41]. Although there are plenty of excuses in support of these odd cravings, junk foods are always labeled as harmful.

The advantages and disadvantages of nonfood cravings have been widely studied, and an additional questioning type of desire is *pica*, consumption of nonfood substances, observed worldwide and posited to be somewhat biologically adaptive and culturally supported [35, 36, 42–44]. In most cases, their reasons are not supported scientifically and it seems to prevent complications related to pregnancy [26, 29–32, 37, 45–48]. Furthermore, the [39] funding concluded that food cravings are intended and consumed by women's beliefs to make certain about baby health status. Enthusiasm to nonfood items affects the absorption of the substance and causes intestinal difficulties that lead to physiological disruptions [36, 42]. These nutritional malpractices result in deficiencies of calories, protein, minerals, and vitamins and cause severe malnutrition in the fetus that can cause changes of structure and metabolism, disproportion, or comparative shortage of nutrients, and may affect the health of women and their babies [27, 32]. Accordingly, culturally mediated food taboos and carvings are contributing to the anemia and growth limitation of intrauterine primary to morbidity and mortality in women and infant [11, 26, 27, 29, 30, 32, 49, 50], and reducing the burden of such malpractice is the main concern in Ethiopia. However, there is a rising tendency in anemia in women, neonates, and infants, especially increasing from 17% in 2011 to 24% in 2016 in mothers [51]. It may also lead to stillbirth [52], premature birth [53], low birth weight [54, 55], and retarded development of cognition in the infant [56–58]. Women from Ethiopia were placed on a slight weight throughout the pregnancy period theoretically for comparable explanations [59]. These mothers have complex prevalence of emergency caesareans as well as interventions in labor time, early hemorrhage of delivery, preeclampsia, pre-/postterm birth, stillbirth, and a little fetus at gestation [52–55, 59, 60]. A nutritional pattern described by a good intake of plant foods, vegetables, and vegetable oils declines the danger of preeclampsia [61].

Dietary counseling, culture, beliefs, educational status, age, attending antenatal care, and multiparous and pregnant women are the most important factors for the occurrence of food taboos during the pregnancy period [9, 26]. Accordingly, treatment of severely malnourished children, nutrition education, and providing of micronutrients to mother and children by enhanced outreach strategy through supplementary food and transitioning into the health extension program, micronutrient intervention, health facility nutrition service, community-based nutrition, and essential nutrition actions are the very growing agenda to improve nutritional practice at the health sector [51].

## 2. Basic Concept of Nutritional Practices

According to [48] and essential nutrition action [62], diet and nutritional practices such as meals from different food groups, changing meal frequency and amount, not skipping meals, and eating at least one additional meal per day are suggested during lactation/pregnancy. In a woman's life cycle, there is no time where nutrition is more important than before and/or during pregnancy [63]. Therefore, pregnant women need to consume healthy diets to ensure superfluous vitamins and minerals, increase their calorie intake, and avoid certain foods and chemicals to optimize the growth and development of their baby along with supporting alterations in maternal tissues and metabolism [64–66]. A healthy diet includes a variety of foods, such as green/orange vegetables, well-cooked meat, fish, legumes, nuts, whole grains, and fruits [67]. Particularly, the need for macronutrients (energy and protein) and several micronutrients including iron, iodine, zinc, magnesium, selenium, folate, vitamin B6, niacin, riboflavin, thiamine, pantothenic acid, vitamin C, vitamin A, vitamin B12, and choline is increased from 6 to 50% in a descending order [64, 65]. Expressly, eating animal food products [68] and additional foods containing protein, perhaps legumes and beans [69], can significantly impact the nutritional status of women. Legumes, particularly beans, are commonly accessible in addition to low cost and therefore currently they are used as a realistic option aimed at this individual.

However, some women often lack access to a healthy diet that provides for their increased nutritional requirements because of some nutritional malpractices which are often practiced in low- and middle-income countries [70–72]. In Ethiopia, there is a shortage of researched data and kinds of literature that explore and focus on the nutritional habits of women. The consequences of poor nutrition to both baby and mother are not recognized by women and indicate the degree of women's awareness about diet, which is not adequate [8]. Understanding regarding the need for improved nutritional intake of lactating women was reported by most participants in West Arsi Zone, Oromia, Ethiopia [45]. It is an imperative drift as women's nutrition during the pregnancy period is significant to reduce complications related to pregnancy such as morbidity/morbidity to mothers and infants. However, to date, very little has been done to assess the general knowledge of Ethiopian women about nutrition during pregnancy [27, 73]. Inadequate serving sizes and skipping meals were also reported by women with the associated reason for low socioeconomic status [37].

Fasting was also practiced during the day of Ramadan by Muslim women as well as exclusion of animal-sourced food items on Wednesdays and Fridays and Easter and Christmas for orthodox women [30, 48]. The study in [73] showed lower knowledge of women (less than 30%) regarding the sources of vitamins, minerals, protein, vitamin A, and iron. It also reported that most women in Ethiopia did not follow a healthy eating style and knew major food groups during the pregnancy period. About 71.2%, 68.6%, and 39.3% of lactating women were not eating meals additionally [37, 45, 47], respectively. This dietary behavior of pregnant and lactating women was also common and further supported by the formative finding of [48]. In another study, limited numbers of women (33.2%) had a change of dieting trends such as increasing frequency of feeding and taking of foods containing extra carbohydrates [30]. According to a study from the northern part of Ethiopia, changes in food intake during lactation were not practiced by most of the lactating mothers [26, 47] but it was better in the study in [45]. Some women are practicing decreasing their dietary intake because of two reasons: the experience of aversion and nausea to different foods in the first three months [48] and the fact that, in the later periods of pregnancy, women deliberately lowered their dietary intakes to have a little fetus and minimize delivery difficulty [32, 48]. The consumption of fruits, fish, meat, and some vegetables during the pregnancy period was low compared to the state of the prepregnancy period [26]. Removal of fish and meat and [74] caffeinated and alcoholic beverages [33] was reported by women during pregnancy to promote their health and their embryo, and it minimizes the introduction of pathogens or toxins. In Ethiopia, the complications of pregnancy outcomes in mothers are moderately elucidated through socioeconomic drawback, deprived education, prenatal carefulness, and health being of less priority; then what additional factors subsidize these complications remains unidentified [27, 29, 31, 59, 73].

### 2.1. The Magnitude and Motivational Drivers of Nutritional Malpractices of Women

Different nutritional malpractices such as taboo of healthy diets, craving of junk foods, and craving of nonfood items (pica) are practiced by women from different divisions of the planet [27, 29, 31, 35, 37, 38, 46, 75, 76].

#### 2.1.1. Food Taboo Practice in Women

From different studies in diverse measurements of Ethiopia, women were forced to remove nutritious foods concerning their habits traditionally and maintain the health of women as well as their babies [26, 29–32, 37, 45–48]. Pregnant women avoid certain foods for a range of reasons associated with pregnancy outcome, the birthing process, and avoiding undesirable visual features in the baby. However, it is reasonably evident that the wide restrictions of healthy diets are further probable to situate the wellbeing of both baby and mother in danger. Accordingly, a study from Awabel District, Ethiopia, reported that 27% of pregnant women practiced taboos for healthy food items. Accordingly, pregnant women believed that the reason for the taboo is that when they consumed banana (35.7%), something is attached to the head of the fetus; pimento (32%) burns the fetus; cabbage (24.3%) disturbs the fetus; sugarcane (44.3%) increases the seminal fluids.

Furthermore, pregnant women avoided groundnut and pumpkin as they consider that these foods enhance fetus weight, which results in difficulty in delivery, linseed causes defeat of fetus strength, and nug modifies the fetus color and creates black color. Porridge (34%), tea (18.7%), coffee (19%), and coca (16.3%) also were not taken with the fear of fetus burning and abnormality and abortion [31]. In another study, different food items such as honey, linseed, nuts, and milk were reported as mostly excluded diets with the feeling of fear concerning abortion, stillbirth, birthing, and baby skin discoloration [30]. Green chili pepper (13.0%), organ meat (15.7%), and dark green leafy vegetables (16.7%) were avoided by the participants [29]. Another finding from the Shashemene district showed that many healthy fruits like pineapples, orange, avocado, and mango were not taken by women [30], which is important to protect babies from exposure to worms, diarrhea, and malaria in their life [77]. Furthermore, various green vegetables were removed from daily intake as taboo [30], with the assumption of bad odors occurrence in both mother and fetus, as well as in preventing a baby from being bald. Taking fruits and eggs together and blending cheese with meat were reported as not good for the mother and fetus [30]. Dairy food products such as yogurt, milk, and cheese were avoided with the cultural misconception of pregnant women in the study of Zerfu and his colleagues [51].

Besides, potatoes and sweet potato [27] and sugar cane [28] were not consumed during pregnancy to prevent the baby from gaining weight and lessen the birth difficulty [27, 30, 76]. Additionally, white dietary items such as milk, porridge, fatty meat, banana, and potato were not included in the daily dishes [76, 77] predominantly to be apprehensive based on the plastering behavior on the fetus's head with white blotches [30, 32]. Likewise, other studies also revealed that good food products (meat and milk) are tabooed and allied with fetus size and the difficulty at the time of labor [30, 32, 76]. Hadush and his colleagues also reported that solid food items were not consumed to lessen the bleeding complication during delivery [32]. Furthermore, women during gestation and lactation are removing foods such as bread, yogurt, milk, cheese, meat, and water [32, 78, 79]. Previously, various studies were reported from different parts of Ethiopia [26, 50] and Kenya [80, 81] to demonstrate taboo behaviors of pregnant and lactating mothers, especially to vegetables and meat.

#### 2.1.2. Food Craving Practice in Women

Food cravings are a strong desire and forceful yearning to get foods that are highly interested in by particular groups of individuals at a specific period. This is commonly termed as a divergent state described by a deep need to find a diet [27, 37, 76]. Across cultures, a craving for items not typically desired is often considered a hallmark of pregnancy. Women are known for craving sweets, fruits, calorie-dense foods, odd combinations such as pickles and ice cream, or pica substances, such as clay and chalk [44, 82, 83]. Despite the widespread occurrence of cravings, this phenomenon remains relatively understudied [83].

Evolutionary theories proposed to explain food cravings include a need to seek foods to either satisfy the energetic demands of the growing fetus or to replenish nutrients lost from nausea, vomiting, and food aversions in the first trimester [84]. Research has also suggested that cravings for nutritional foods could operate as a buffering strategy to store nutrients in environments that lack adequate access to resources. While the biological and environmental correlates are essential to investigate, food cravings also could serve a psychosocial function. Cravings that follow the period of nausea and vomiting in the first trimester might function to replenish lost nutrients [82]. By the tenth week of pregnancy, women begin to store excess fat, reduce physical activity, and consume foods with high calorie content to support fetal growth and development [85]. Meat, egg, and vegetables are the most common food items craved by women because of their flavor and color [37]. Another study also agreed that foods such as chips, chocolate, pickles, citrus fruits, and ice cream were regularly craved by pregnant women [86]. The most craved food items are sweets, particularly ice cream, vegetables, and fruits; nevertheless, tropical fruits and watermelon are termed more regularly than citrus fruits [33]. Another study also noted that fruit juices, fruits, and sweets are craved food items [39], while carbohydrates (pizza), sweets (chocolate), and animal protein (steak) are the most craved diet [87]. This is significant to monitor healthy food consumption patterns during the pregnancy period to encourage the outcomes for mother as well as for infant; however, the intake of unhealthy craved dietary substances causes the development of difficulty in maintaining good outcomes of pregnancy [33].

Different studies have shown that high desirability to low-nutrient dense foods (foods high in energy-like sweets) could be described by the promoting market campaigns, foods' low cost, and availability [88]. Unhealthy dietary patterns, together with the increased consumption of processed foods rich in simple sugar and sweeteners, are some of the reasons, among others, for the increase in obesity rates during the last years [60]. Henceforth, the craving for vegetables as well as fruits by closely as numerous womenfolk, despite its cost and less advancement, is an encouraging result of the finding. One hypothesis may be that sweets are higher in calories so that cravers might achieve a satiety level more quickly, while the fruits and vegetable groups of cravers did not feel full as rapidly after eating fruits and vegetables. Therefore, fruits and vegetable cravers may have the interest to look for another source of oral satisfaction, herein sample, by mainly chewing proceeding ice [33]. This study also explains the point of hormonal activities that occur at the midpregnancy period to normalize the movement of glucose in the developing fetus to the triggering mechanism, especially for sweets eaten as craved foods.

#### 2.1.3. Pica Practice in Women

Despite the pica activity experienced and a thoughtful desire aimed at substances that are mainly nonnutritive materials (clay, paper, metal, soil, chalk, glass, and sand), the situation is predominantly surrounded by pregnant women through Sub-Saharan African countries, like Ethiopia, Ghana, Kenya, Rwanda, Tanzania, Nigeria, and South Africa [35, 37, 38]. This one is highly associated with using medical cure, psychic manner, ritualistic behavior, and prolonged hunger, traditional medicine, customary social activities, and traditional customs [38]. The popularity of pica behavior strongly differs through the different study areas and 68%, 45.6%, 30.6%, 27%, and 59% were reported, respectively [33, 36–38, 42]. Consumption of soil (sand, clay, and wall mud), ash, residuals of coffee, white soft stone, old dried animals, and green leaf of coffee was reported in pregnant women from various parts of Ethiopia as a minimum of once per day because of interest of individual and the substance's smell [37]. The degrees of these malpractices and eating patterns are significantly fluctuating from a unique community towards a new community. Nevertheless, food aversion is largely more corporate amid less educated and rural communities [50, 89].

Food taboos were found to be dominant among remote rural residents with little access to nutrition and health services, older, and uneducated women [26, 90–92]. In such conditions, cultural philosophies, whether factual or immoral, are inclined to shape activities. Food avoidance with misconception was expressively associated with the way of origin, especially in the Ethiopian context; Oromo cultural communities were revealed to obligate several dietary taboos in comparison with Wolayta cultural communities [30]. These nutritional practices are highly prevalent among women who were less educated, younger women, and those who come from lower socioeconomic circumstances [76]. Pregnant women who have uneducated husbands are further exposed to escape foods. The expected explanation could be the fact that husbands with a good education can contribute and give support to the women not to avoid nutritious diet during pregnancy period. Also, the growing activity of potatoes, sweet potato, and coffee was considerably associated with nutrition aversion of women during pregnancy [37]. It could be explained by the fact that the accessibility to cultivated products increases the chance to acquire another food commencing the market through selling the farmed products. Therefore, women might be avoiding the food items which are disliked by them and they can take what they have chosen [37].

Furthermore, previous antenatal care and age of the mother were significantly associated with food taboo. [31]. Several findings have shown that education, age, and socioeconomic prominence are related to awareness regarding the significance of a well-adjusted diet throughout the pregnancy period [30, 73, 76, 93]. Frighteningly, this high glassy food aversion during pregnancy eliminates the entire groups of food and causes lower dietary diversity. Accordingly, the common food items consumed by women are incomplete and limited to Shiro wot (flour of chickpea), teff injera (flatbread sourdough), kocho (fermented inset and flatbread), and bread of wheat, which are mostly rich in carbohydrates [76] and barren of additional important nutrients (proteins, vitamins, fats, and minerals) that have a vigorous role to optimum functioning of body and growth and development of fetus during pregnancy. Eating extra meals during the pregnancy period was statistically related to food avoidance. Accordingly, pregnant women who were taking an additional occasional pattern had 1.62 times added behavior of food aversion than women who were not taking an extra meal [37].

## 3. Potential Health Consequences of Nutritional Malpractices

Malnourishment is a substantial health difficulty disturbing voluminous women in Ethiopia and it has thoughtful significance for gestational and delivery outcomes [30]. Gravidity, by itself, affects pregnant women to be vulnerable to undernutrition because of the biological intensification in nutrient response, which could not be sufficiently encountered by dietary consumption.

### 3.1. Adverse Effect of Taboo Practice

Food aversions are then indistinguishably associated with the nutritional status of mothers, impacting their health as well as the wellbeing of their babies during the pregnancy period in Ethiopia [27]. In most cases, poor dietary intake among reproductive-aged mothers disturbs the probabilities women endure in addition to the health of the child [5]. As a result, additional hampering of diets attributable to pregnancy-associated food aversions and mythologies may extremely distress the wellbeing of the women in addition to the health of the fetus [50, 80]. About 7% of physical and mental disabilities, predominantly sight damage and limb deformity, are held to be triggered by betrayal diet taboo. The major problem of food taboos is preventing pregnant women from accessing a well-balanced diet, resulting in a high prevalence of low birth weight and harm to mother and baby [94].

Mothers who practiced customary food taboos during pregnancy have better chances of emerging a variety of adverse pregnancy consequences. The massive limitation of diet substantially controls the consumption of vigorous nutrients essential for optimum maternal condition and fetal growth [30]. Evidence displays higher proportions of taboo, keeping out the eating of food items rich in iron conforming legumes, meat, and green dark vegetables which can cause anemia during pregnancy and also to nonpregnant women [95]. These traditionally transmitted behaviors and misunderstandings result in reduced dietary value and mixture, which may subsequently lead to developing deficiencies on a range of micro- and macronutrients. [26, 73, 80, 96]. These nutritional insufficiencies are significantly associated with many argumentative pregnancies as well as developmental consequences (intrauterine growth restriction and anemia) leading to the direction of infant and maternal mortality and morbidity [26, 30, 49, 73, 80].

Preceding studies completed in Ethiopia informed the presence of universal food restrictions, which might be causative to the problem of anemia in women [26, 27, 29, 32, 50, 95]. The occurrences of anemia at the time of pregnancy take and are associated with a series of opposing outcomes [52]. for example, a larger threat of infection, bleeding, preeclampsia stillbirth, and intrauterine growth restriction [52], early birth [53], low birth weight [54, 55], and retarded development of cognition among infants [56–58].

### 3.2. Adverse Effect of Unhealthy Foods Craving

Consumption of junk food is also harmful to both mother and the baby. Junk food does not have any fiber that can result in uncomfortable bowel moments that may rupture the fetal bag and cause premature birth [63, 97]. Besides, of particular relevance here, recent researchers have identified food cravings as possibly having a role in excess gestational weight gain [33, 40, 98]. Accordingly, overconsumption of certain nutrients (such as protein, calories, or fat) leads to excess gestational weight gain that could have an adverse consequence on the mother as well as on the infant later in life [33, 40, 60]. The consequences can be pregnancy-induced hypertension, gestational diabetes, and various birth defects which undermine their physical and psychological wellbeing during pregnancy and increase their chance of delivering macrosomic babies (babies born with a birth weight greater than 4 kg) [41, 99], who may also develop obesity [41, 100] and diabetes [41] later in life and low birth weight [60, 101]. Another drawback of weight gain during pregnancy is that overweight/obese women usually have greater difficulty initiating breastfeeding and shorten the duration of breastfeeding due to feelings of discouragement [86, 102]. Sugar-rich foods worsen pregnancy discomforts (such as nausea, vomiting, constipation, and heartburn), increase weight, contribute to gestational diabetes, preterm labor, and preeclampsia, and increase the risk of metabolic syndrome in babies [60, 63, 97]. The safety of artificial sweeteners, including aspartame and saccharin, is controversial, as some health practitioners believe that they are safe if used in moderate amounts, while some others differ. Accordingly, aspartame can lead to birth defects and saccharin is known to remain in the fetal tissues [103].

Too much salt is not good during pregnancy as it can lead to water retention in the body, resulting in swelling in the feet/hands and blood pressure in pregnancy [63, 104]. It also causes a massive development of adult hypertension, and renal and coronary heart disease may happen by intrauterine exposure to poor nutrition such as nitrate-rich foods that contain sodium and saturated fats [63]. Additionally, the nitrates turn to nitrosamines in the bodies, increasing the chances of cancer in mothers and abnormalities in the fetus [63].

Women also have cravings and eating of smoked and refrigerated seafood which contains *Listeria monocytogenes* bacteria and causes listeriosis (associated with symptoms such as diarrhea and vomiting) that could lead to illness in newborns and even miscarriage or stillbirth [101, 104]. Many types of research say that pregnant women who eat doughnuts, chips, oily foods, and candies too much have a risk of defective childbirth; basically, the baby's brain and the mother's brain react differently [63].

Higher desire for raw sprouts is highly prone to *Listeria*, *Salmonella*, and *E. coli* bacteria and leads to premature birth, miscarriage, stillbirth, infections in newborns, and severe illnesses [63, 101]. Overcraving for fish such as shark, swordfish, king mackerel, and tilefish allows mercury positioning which is a neurotoxin and is linked to brain damage, child's heart failure, improper growth, and developmental delays in babies [63, 104]. Higher amounts of caffeine could increase the chance of miscarriage, premature birth, low-birth-weight babies, and withdrawal symptoms in infants [101, 104]. The lining of the food cans contains Bisphenol A, a toxic substance that affects the fetal endocrine activity and causes fertility problems, cancer, liver ailments, and heart diseases in pregnant women and the tinned foods might be too old to eat and harbor harmful bacteria due to their long shelf-life [63].

### 3.3. Adverse Effect of Pica Practice

Geophagia remained associated with utilizing iron exhaustion and malabsorption of crucial nutrients [105]. Above and beyond, a finding from Tanzania showed that the change in average hemoglobin level via pica actions in the course of pregnancy was significant in statistical analysis. This result also established that geophagy and amylophagy are situated to the natures of pica material, which are intensely associated via deficiency of iron, as well as certain gastrointestinal indispositions for instance constipation, abdominal pain, and diarrhea [44]. Furthermore, ingested soils reduced the absorption of bioavailable nutrients especially iron, copper, and zinc which are already available in the foods [44, 106]. Another study also showed that pica practice is an inhibiting factor of hemoglobin levels [107]. Other proposed causes of pica are gastrointestinal difficulties, reaction to stress, hunger, and cultural belief [108]. Accordingly, eating such nonfood items could enhance the deficiency of calcium/iron/zinc, allows for the introduction of toxic compounds/quantities of nutrients not tolerated in disease states, and reduces the intakes of nutritious foods leading to inadequate dietary intake of essential nutrients [11, 42, 69, 109–111].

On micronutrient deficiency, some studies have reported association of pica with increased anemia, low plasma zinc level, low hematocrit, and low hemoglobin. This cannot completely explain whether or not pica is related to micronutrient deficiencies, but it does imply that pica is a risk for these deficiencies, all of which affect the health and wellbeing of an individual [11, 110, 111].

Several of these destructive pregnancies, as well as birth consequences, can be there to improve popular encouraging of women to upturn the diversity of diets. Pica practice is innocently associated with way of undernutrition of pregnant mothers [37]. This capacity can be the repetition of pica ingredient interference by the absorption of significant nutrients. The equivalent study piloted in Kenya indicated that performing pica material may inhibit the absorption of the nutritious ingredient and affect intestinal obstacles [42]. Zinc deficiency is another problem for Ethiopian pregnant women [68], which is significantly related to delivery's preterm as well as birth with lower weight [112]. As for iron deficiency, high reliance on cereal-based foods and low intakes of pulses and animal proteins are the main contributors to this problem [69]. Nevertheless, the nutritional status of women stays fundamentally unknown; women from African origin are the most likely to be anemic in addition to being at bigger risk of child mortality and morbidity [109].

## 4. Conclusions and Recommendations

This review found pieces of evidence that nutritional malpractices have a significant influence on women's nutritional status, maternal mortality, and poor fetal outcomes. Maternal nutrition is very important for childbearing-age women, especially to make adjustments in maternal body composition, metabolism, and function of various physiological systems. This should be further maintained through improved dietary intakes, intrahousehold food distribution, health/hygienic care, and healthy nutritional habits as well as women empowerment. Despite this, cultural misconceptions, a strong desire to unhealthy diet, and backgrounds can significantly control the action with real food consumption of women, which ties into the availability of foods and preferences of food and the chance of evolving cravings and taboos. These nutritional malpractices are common among pregnant and lactating women, indicating guarding the wellbeing of women, fetuses, and babies.

Food taboos are strong food aversion items because of the culturally transmitted background and fearing behavior of women like plastering on the fetal head, the colors of the fetus, burns of the fetus, birth difficulty, fat baby, fear of abortion, and stillbirth. On the other hand, different unhealthy and nonfood items are craved by women due to their interest, flavor, taste, color, and other olfactory satisfactions. Furthermore, food craving can be related to change in physiology/hormonal levels, as a response to elevated nutritional needs, cultural factors, and the presence of a specific desired ingredient in the craved food. Such a consumption pattern hurts the outcomes of the pregnancy. These include short- and long-term complications for mothers (pregnancy-induced hypertension, gestational diabetes mellitus heart disease, and cancer), discomforts, preterm labor, preeclampsia, and intrauterine growth restriction, and offspring (macrosomia, trauma at birth, stillbirth, low birth weight, obesity, hypertension, cancer, diabetes, metabolic syndrome, renal disease, decreased fetal growth, heart failure, and poor cognitive development). Recognizing that nutritional cravings remain real is important both for mothers and health professionals. In another way, cravings for nonfood items (clay, sand, wall mud, coffee residue, soft white stone, ash, old dried animals like oxen, cows, and skin, and green coffee leaf) interfere with the absorption of important nutrients (iron and zinc) causing intestinal obstructions, negative pregnancy, and birth outcomes.

The scope of these malpractices can vary greatly by time, community, culture, norm, religion, residency, season, and dietary practices. Still, it can be associated with socioeconomic status, nutrition information, health care/facilities, social support, and level of awareness. As a general rule, this literature review concludes that nutritional malpractices are strongly affecting the daily consumption of protein, energy, vitamins, and minerals. Indeed, the deficiencies of those nutrients are associated with some adverse pregnancy and developmental outcomes such as anemia and intrauterine growth restriction, leading to greater maternal and infant morbidity and mortality. Additionally, poor diet quality and diversity are significantly associated with a range of adverse outcomes such as a greater risk of infection, preeclampsia, bleeding, stillbirth, premature birth, low birth weight, and poor development of cognition in the infant. Therefore, there is a need for women who are pregnant or are of childbearing age to look for professional nutrition education, counseling, and awareness generation programs to learn how to adopt healthy dietary habits. This can be improved by empowering health practitioners (health extension workers, doctors, dietitians, and others) in providing effective nutrition information that should be explored and given the overburdened public health system. Furthermore, there is a need to put mechanisms that can routinely identify women observing nutritional malpractices, assess the reasons, and provide appropriate nutrition education. Other governmental and nongovernmental organizations and various public associations such as women's associations should also be actively concerned to make a negligible extent of these culturally transmitted and harmful nutritional habits from the different living environments. Women should be encouraged to eat up during pregnancy and lactation and to provide supplementary food to poor women who cannot afford it. Besides, the government of Ethiopia should design health and nutrition education programs that could capture the cognizance of the popular beliefs and misconceptions regarding food during pregnancy and use innovative means to minimize their negative and maximize their positive nutritional effects, a long-term solution to the problem.

## Data Availability

This is a narrative review study. All relevant data are presented in the manuscript. However, any required further information can be provided by the corresponding author.
